# Cause of Typhoid Fever

**Published:** 1890-01-25

**Authors:** 


### The Practitioner's Outlook and Retrospect.

[*The Editor will be glad to receive offers of co-operation and contributions from members of the profession. There is no doubt that much valuable material full of interest and instruction is at present lost to science. This is largely so in the case of* (1) *the younger and less-known members of the hospital staff,* (2) *those connected with cottage hospitals, and* (3) *the great body of medical practitioners not attached to any institution. The co-operation of all is cordially invited to enable the Editor to make* The Hospital *of the utmost possible use and value to the profession. Specialists willing to review books on their special subjects are invited to communicate this fact at once. All letters should be addressed to* The Editor, The Lodge, Porchester Square, London, W.]

### MEDICINE.

### CAUSE OF TYPHOID FEVER.

Dr. W. Thorton Parker, of Newport, has an article in the *New York Medical Record* of December 7 last on one of the causes of typhoid fever. He protests against the claim that given a case of typhoid fever the bacillus typhosus must have had some influence in its causation. That many different-shaped bacilli can be found in the discharge of patients suffering from various disorders there is no doubt, but Dr. Parker does not attribute typhoid to the emigration of a particular bacillus. It is difficult for him to believe that there is a bacillus for typhoid, another for cholera, another for diphtheria, another for scarlet fever, another for phthisis, etc. More easily believed and understood is the idea of the "unity of poison." Fœcal odour alone can and often does develop typhoid fever. Dr. Parker believes that the time will come when the remarkable exploits of the many-shaped and many-named bacilli will be one of the curiosities of medical literature.

